# Prognostic significance of Hypoxia-Inducible Factor 1 alpha(HIF-1alpha) expression in serous ovarian cancer: an immunohistochemical study

**DOI:** 10.1186/1471-2407-8-335

**Published:** 2008-11-16

**Authors:** Alexandros Daponte, Maria Ioannou, Ilias Mylonis, George Simos, Marcos Minas, Ioannis E Messinis, George Koukoulis

**Affiliations:** 1 Department of Obstetrics & Gynaecology, University of Thessalia, Larissa, Greece; 2 Department of Pathology, University of Thessalia, Larissa, Greece; 3 Laboratory of Biochemistry Department of Medicine and Institute of Biomedical Research and Technology (BIOMED), Larissa, Greece; 4 Department of Hygiene and Epidemiology, University of Thessalia, Larissa, Greece

## Abstract

**Background:**

The hypoxia-inducible factor (HIF) has emerged as an attractive target for cancer therapy. The few publications addressing the prognostic significance of Hypoxia-Inducible Factor 1α (HIF-1α) cellular expression in ovarian cancer produced contradictory findings which are not permissible to widely acceptable conclusions and clinical applications. Our study was designed to investigate this by including a comparatively large number of cases and by using a combination of antibodies to analyze immunohistochemically the expression of HIF-1α.

**Methods:**

One hundred (n = 100) neoplastic and 20 benign (controls) pathological samples from paraffin-embedded tissue were included. They were classified after surgery as stage I (n = 23) and stage III G3 (n = 55). Also 22 borderline serous adenocarcinoma patients and 20 benign controls were stained. The mean follow up was 3 years. Only patients with the diagnosis of serous carcinoma of stage III, G3 who received 6 cycles of postoperative TC (175–180 mg/m2 paclitaxel and carboplatin after calculating the area under the concentration curve) with complete medical records (n = 55) were selected for survival analysis. The survival analysis of the samples compared two groups after the patients were dichotomized by HIF-1α final score to positive and negative.

**Results:**

The frequency of the nuclear expression of HIF-1α in benign tumours was significantly lower (median: no expression) than in borderline and ovarian cancer tumours combined (p < 0.001). HIF-1α expression in serous ovarian carcinoma was not stage dependent. The overall survival of patients with tumours that stained strongly for HIF-1α was significantly shorter than that of patients with tumours that stained weakly or were negative for HIF-1α (p = 0.01). Kaplan-Meier survival curves confirmed that HIF-1α "positive" had decreased overall survival compared to HIF-1α "negative" patients (p = 0.003) and this was an independent adverse prognostic factor (multivariable analysis p = 0.006). HIF-1α "positive" patients displayed a shorter median progress free interval (PFI) (not statistically significant p > 0.05). Interestingly the overall PFI of the subgroup of patients that have undergone suboptimal cytoreduction at primary surgery (n = 21) with tumours that stained strongly for HIF-1α was significantly worse than that of patients with tumours that stained weakly or were negative for HIF-1α (p = 0.03).

**Conclusion:**

Our report confirms the prognostic value of HIF-1α when restricted to poorly differentiated serous ovarian carcinoma. In addition it shows that this association is elusive, since it is not only methodology-related but it can be antibody-depended. There is adequate evidence to speculate that targeting HIF-1α could improve the long-term prognosis of these patients In order to increase the overall sensitivity of the immunoassay, maintaining acceptable levels of specificity, a panel of antibodies should be used.

## Background

The hypoxia-inducible factor (HIF) is an alpha (α)/beta (β) heterodimeric DNA binding complex and directs an extensive transcriptional response involving the induction of genes relevant to tumour progression, such as angiogenesis, glucose/energy metabolism, cellular growth, metastasis, and apoptosis [1,2]. HIF-1α has emerged as an attractive target for cancer therapy [3,4].

HIF-1α protein expression in ovarian cancer was first investigated using immunohistochemistry (IHC) by Zhong [5]. Thereafter, HIF-1α protein overexpression was shown in 54–69% of the cancerous specimens tested vs. 12.5–31.4% in non-cancerous ovarian tissues [6,7].

Birner investigated for the first time, by IHC, the relationship of hypoxia-inducible factor 1α (HIF-1α) expression with prognosis and on response to chemotherapy in epithelial ovarian tumours. He concluded that HIF-1α protein overexpression alone has no impact on the prognosis of ovarian cancer, whereas in a subgroup of patients with concurrent overexpression of 1α and p53 protein, a significantly shorter overall survival was observed [6].

In a subsequent study, using RT-PCR, the expression level of 1α had no relation with the survival of ovarian cancer patients and it was also independent of age, clinical stage and histological subtype [8].

However, two recent publications offered additional information indicating a relationship between HIF-1α expression and prognosis in ovarian carcinomas.

Nakai by using Western Blotting concluded that in 52 patients with sub-optimally resected stage III/IV tumours and further treated with combination postoperative chemotherapy Taxol, Carbo (TC), HIF-1α expression correlated with significantly better survival [9].

Osada in a recent IHC study (24 patients with ovarian carcinoma stage III/IV patients, 10 of them poorly differentiated G3, followed by cisplatin-based chemotherapy) showed that nuclear HIF1a immunoreactivity was an independent marker of poor prognosis [10].

At this point, some of the findings appear to be contradictory and not permissible to widely acceptable conclusions and clinical applications. There may be several factors responsible for the published data variability. Our study was designed to address two of them by including a comparatively large number of cases and by using a combination of antibodies to analyze immunohistochemically the expression of HIF-1α.

## Methods

### Patients and samples

One hundred (n = 100) neoplastic and 20 benign (controls) pathological samples from paraffin-embedded tissue were included. The explorative laparotomies, chemotherapies and the treatment of patients included in this trial was carried out in the University Hospital of Larissa-University of Thessalia Greece. All the necessary informed consents as requested by the Institutional Review Board (IRB) of the University Hospital were given. They were classified after surgery as stage I (n = 23) and stage III (n = 55), G3 and 22 borderline serous adenocarcinoma patients and 20 benign controls. The mean follow up was 3 years. At the time of the analysis, 13/55 were deceased due to their disease. Due to the short follow up all stage I and borderline patients were alive at the time of the analysis. Fifty five stage III pathological samples from paraffin-embedded tissue were included in the survival analysis. Only patients with the diagnosis of serous carcinoma of stage III, G3 who received 6 cycles of postoperative TC (175–180 mg/m2 paclitaxel and carboplatin after calculating the area under the concentration curve (AUC) with complete medical records were selected for survival analysis. All above-mentioned samples were obtained one per patient. Clinicopathologic information was obtained from medical records.

Cancer patients were classified after a staging laparotomy was performed (the most common initial surgical procedure consisted of abdominal hysterectomy, bilateral salpingo-oophorectomy, omentectomy, and lymph node sampling). The surgery was classified as complete when < 1 cm of tumor was left behind.

All slides were reviewed by two pathologists (G.K, M.I). The surgical procedures were carried out in the Department of Obstetrics and Gynaecology University of Thessalia.

In order for a sample to be given a "positive" final score convincing and easily detectable nuclear staining should be seen with at least 3 out of the 5 HIF-1α antibodies tested. Otherwise it was classified as negative.

The stage, grading, histology, age, family history, Ca 125 and level of cytoreduction (complete versus incomplete) were reported.

Different regimens were given as second- and third-line therapy, and the different groups (after further classifying according to second-line chemotherapy) had small numbers to be analyzed separately. Therefore, although both the progress-free interval (PFI) and the overall survival were calculated, we report PFI after first-line chemotherapy (TC) as indicative of the response to the first-line chemotherapy. The survival analysis of the samples compared two groups after the patients were dichotomized by HIF-1α final score to positive and negative.

### Immunohistochemical staining

Immunostaining was performed with the antibodies listed in Table 1. The tissue samples had all been fixed in 10% buffered formalin, processed and embedded in paraffin routinely. Sections were cut at 3 μm using a Leica TP1020 microtome and dried overnight at 60°C. After deparaffinization in xylene, the sections were rehydrated in decreasing ethanol solutions and incubated in 0.3% hydrogen peroxide for 10 min, to block endogenous peroxidase.

**Table 1:** Antibodies to HIF-1α used in this study*

**Antibody**	**Optimal dilution**	**Source**
H1a67	1:200	Abcam
Rabbit Polyclonal	1:200	Santa Cruz
54/HIF-1α	1:20	BD Biosciences
H1a67	1:75	Neomarkers
Rabbit polyclonal	1:200	Univ. Thessaly

Different methods of antigen retrieval were tested in pilot experiments (data not shown). Under the conditions of the study, optimal antigen retrieval was achieved by microwaving tissue sections in 0.01 M citrate buffer solution (pH 6) for 20 min, (LG WAVEDOM, 850 Watt). This antigen retrieval remained optimal irrespectively of the primary antibody type applied. After the antigen retrieval, the sections cooled and washed in phosphate-buffered saline (PBS) for three times. Tissue sections were incubated overnight at 4°C with each antibody. The optimal dilutions were determined with pilot experiments (data not shown) see Table 1. Then, the slides were washed in PBS and Envision fluid (polymer-peroxidase method, EnVision+/HRP, DAKO, Denmark) was added, followed by incubation for 30 min. Bound antibodies were visualized by using 0.05% 3,3'-diaminobenzidine solution (DAB solution, DAKO). Finally, sections were counterstained with hematoxylin and mounted in Entellan (Merck, Germany). A detailed description of the University of Thessaly antibody has been included in a recently published HIF-1α separate study [11].

### Statistical analysis

Group comparisons were based on Spearman's test for nominal variables (age, Ca125) and on the linear x^2^-test for ordinal variables (stage, grading, complete/incomplete cytoreduction). The HIF-1α scores of the different groups of patients were compared using the non-parametric test for multiple comparison (Kruscal Wallis-Mann Whitney) followed by Dunn's test, which generalizes the Bonferroni adjustment.

The factors possibly influencing the PFI and survival (age, Ca 125, stage, HIF-1α scores) were determined by binary logistic regression analysis using forward likelihood ratio method.

From 55 poorly differentiated serous ovarian carcinomas, 33 tumour samples were classified as HIF-1α protein "positive" (60%%) and 22 as HIF-1α protein "negative" as they did not stain at all (40%). After the patients were dichotomized by HIF-1α expression final score as positive or negative (used as gold standard) Cohen's kappa *κ *statistics were used to evaluate the measure of agreement kappa (*κ *value) for HIF-1α protein detection between the different antibodies.

Uni-variable and multivariable analyses were performed. The covariates age, Ca 125, HIF-1α scores were used for the multivariable analysis (in the 55 stage III, G3, serous adenocarcinoma patients). Progression-free survival and overall survival were estimated by Kaplan and Meier's method. The log-rank test was used to compare differences between survival curves. The Cox regression was used to calculate hazard ratios [12].

These were materialized using GraphPad Prism version 5 and SPSS version 15 software. All P values calculated are two sided. P < 0.05 was considered to be significant.

## Results

HIF-1α protein expression, (nuclear unless otherwise specified), was shown in 14% of the non-cancerous ovarian samples, in 53% of the borderline and in 60% of the cancerous samples tested. The frequency of the nuclear expression of HIF-1α in benign tumours differed significantly (median: no expression) than in borderline and ovarian cancer tumours combined (p < 0.001). The frequency of the nuclear expression of HIF-1α in carcinomas was higher than that of borderline tumours, but this difference was not significant (p > 0.05).

**Figure 1:**
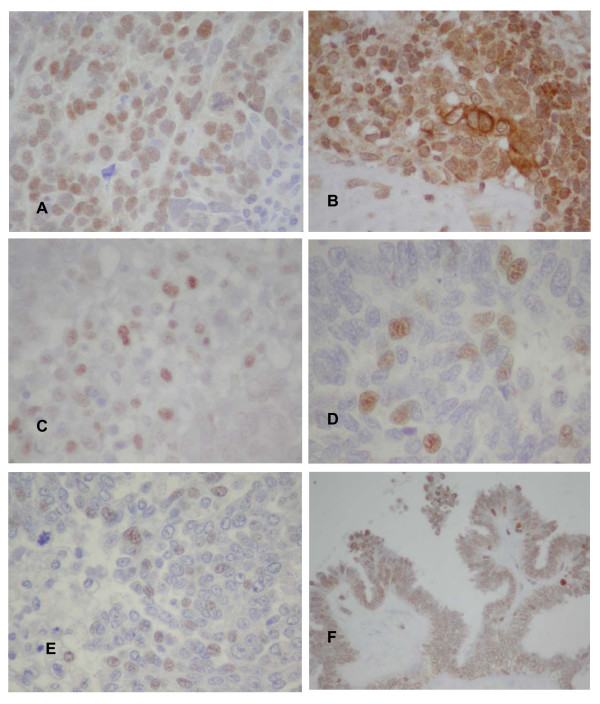
****A **– Serous carcinoma.** Immunostaining for HIF1α with polyclonal antibody made in the University of Thessaly. **B **– Serous carcinoma. Immunostaining for HIF1α with rabbit polyclonal antibody (Santa Cruz).note variable cytoplasmic staining in addition to the variable nuclear staining. **C **– Serous carcinoma. Immunostaining for HIF1α with monoclonal antibody 54/HIF-1α (BD Biosciences). Note focal nuclear staining and lack of cytoplasmic staining. **D **– Serous carcinoma. Immunostaining for HIF1α with monoclonal antibody H!a67 (Neomarkers). Note focal nuclear staining and lack of cytoplasmic staining. **E **– Serous carcinoma. Immunostaining for HIF1α with monoclonal antibody H1a67 (Abcam). Note focal and weak nuclear staining and lack of cytoplasmic staining in this case. Cytoplasmic staining was seen in other cases. **F **– Serous tumor of low malignant potential. Note weak staining in several nuclei and focal intense staining of the nuclei at the tip of the papilla (see arrow and insert). Immunostaining with clone H1a67 (Abcam).

In the subset of 55 poorly differentiated serous ovarian carcinomas the average patients' age was 62.5 and there was no statistical difference between HIF-1α positive and negative patients in regard to age, Ca 125, and complete/incomplete cytoreduction (p > 0.05). Thirty three (33) tumour samples were classified as HIF-1α protein "positive" (60%%) and 22 as HIF-1α protein "negative" as they did not stain at all (40%). Representative immunohistochemical findings are shown and commended in Figure 1.

HIF-1α expression level was independent of clinical stage (p > 0.05).

### Relationship between HIF-1α protein expression and survival in patients with serous stage III poorly differentiated adenocarcinoma

**Figure 2:**
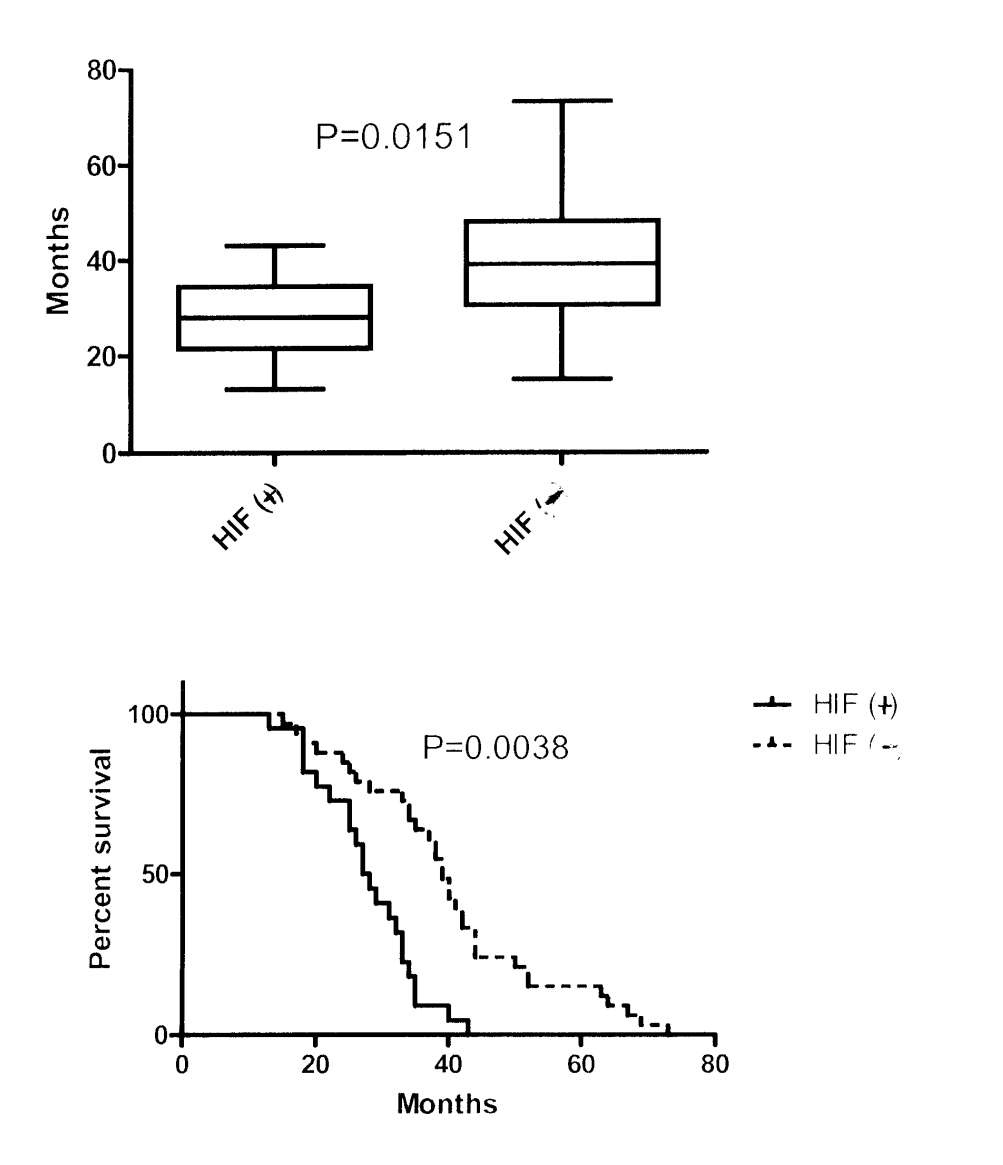
Survival analysis HIF(+) vs HIF (-).

In the 55 patients with stage III, G3 serous carcinomas, the overall survival of patients with tumours that stained strongly for HIF-1α differed significantly than that of patients with tumours that stained weakly or were negative for HIF-1α (p < 0.05). HIF-1α positive patients displayed a median survival of 28 months (18–43 months) versus 39 months (range 15–73 months) for HIF-1α negative patients (Figure 2). Additionally, Kaplan-Meier survival curves confirmed that HIF-1α positive stage III, G3 patients had decreased overall survival compared to HIF-1α negative patients (p < 0.01). Cox regression analysis demonstrated that HIF-1α protein had a hazard ratio (HR) of 3.853 (1.544 to 9.614) (Figure 2). Additionally increased HIF-1α protein expression was an independent adverse prognostic factor for survival (see multivariable analysis in Table 2, p < 0.01).

**Table 2:** Multivariable Analysis for Hif-1a expression for Survival, PFI and in patients with incomplete surgery

	Coefficient	P
Multivariable Analysis for Survival		
Age	-0.57702	> 0.05
**HIF**	**20.42544**	**< 0.01**
Ca125	0.00316	> 0.01
constant	61.99826	
Multivariable Analysis for PFI		
Age	-0.60288	> 0.05
**HIF**	**14.14634**	**> 0.05**
Ca125	0.00606	> 0.05
constant	49.06490	
Multivariable Analysis for PFI (-) incomplete		
Age	-0.07887	> 0.05
**HIF**	**13.56907**	**0.05**
**Ca125**	**0.00606**	**< 0.05**
constant	8.71984	

Multivariable Analysis for Survival

### Relationship between HIF-1α protein expression and progression-free interval (PFI) in serous stage III poorly differentiated adenocarcinomas

HIF-1α positive patients displayed a median PFI of 19 months (range 6–67 months) versus 20 months (8–42 months) in HIF-1α negative patients (not statistically significant, p > 0.05). Additionally, Kaplan-Meier survival curves showed that HIF-1α protein positive stage III, G3 cases had a decreased overall PFI compared to HIF-1α negative patients but this was not statistically significant (p > 0.05). Cox regression analysis demonstrated that HIF-1α had an HR of 1.185 (0.5617 to 2.499). Increased HIF-1α protein expression was not an independent adverse prognostic factor for PFI (see multivariable analysis in Table 2, p > 0.05).

### Relationship between HIF-1α protein expression and PFI in serous stage III poorly differentiated adenocarcinoma patients with suboptimal cytoreduction

**Figure 3:**
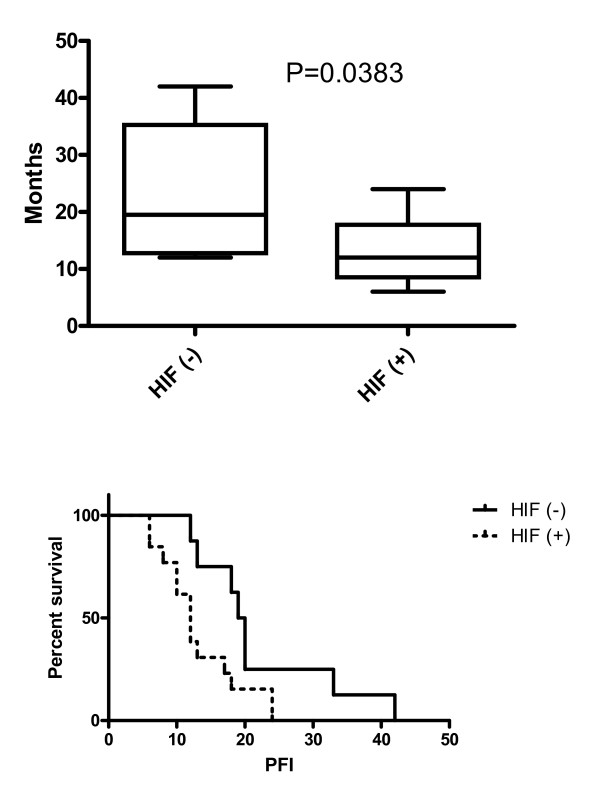
Progress free interval) PFI**_**survival analysis in HIF(+) vs HIF (-) patients with incomplete cytoreductive surgery.

We performed a separate analysis in patients (n = 21) that have undergone suboptimal cytoreduction at primary surgery. The overall PFI of this subgroup of patients with tumours that stained strongly for HIF-1α differed significantly than that of patients with tumours that stained weakly or were negative for HIF-1α (p < 0.05). HIF-1α positive patients displayed a median PFI of 12 months (range 6–24) versus 19.5 months (12–42) in HIF-1α negative patients. Additionally, Kaplan-Meier survival curves showed that HIF-1α positive stage III, G3 patients had a decreased overall PFI compared to HIF-1α negative patients, and this was statistically significant (p < 0.05) (Figure 3). Cox regression analysis demonstrated an HR of 2.88 (1.007 to 8.37) for HIF-1α. Increased HIF-1α expression was an independent adverse prognostic factor for PFI for these group of patients (see multivariable analysis in Table 2, p = 0.05).

### Relationship between HIF-1α nuclear or cytoplasmic expression and PFI and survival in serous stage III poorly differentiated adenocarcinoma

The group of HIF-1α positive samples (n = 33) were further subdivided according to the presence of nuclear or cytoplasmic immunostaining. The patients with samples showing combined nuclear and cytoplasmic HIF-1α staining (n = 14) displayed a median survival of 38 months (24–73) and a median PFI of 18 months (6–28). The ones without cytoplasmic staining and with nuclear only staining (n = 19) displayed a median survival of 39 months (15–69) and a median PFI of 24.5 months (6–67). When these groups were compared, they did not differ significantly for PFI (p > 0.05) or survival (p > 0.05).

### Relationship between HIF-1α nuclear staining with different antibodies in serous stage III poorly differentiated adenocarcinoma patients

**Figure 4:**
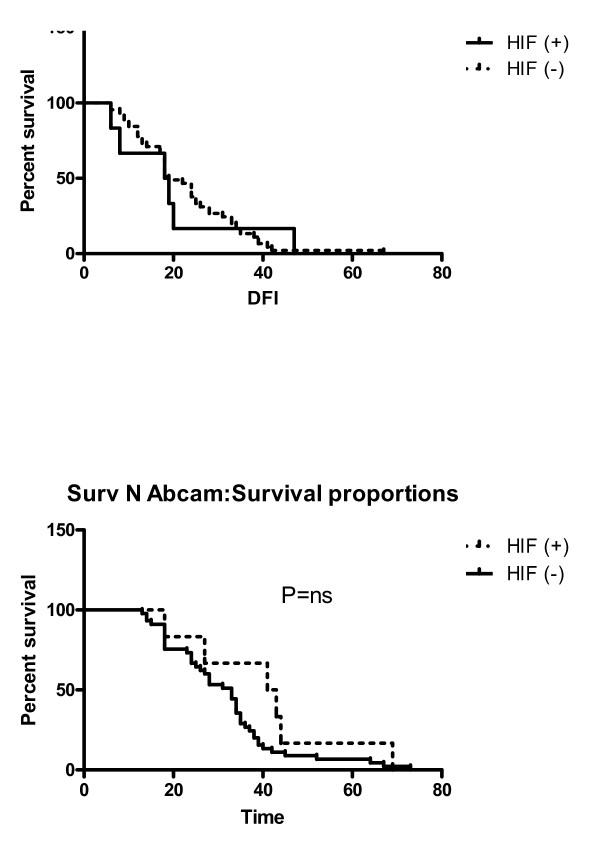
Progress free interval and overall survival curves (p > 0.05) using only Abcams antibody to define HIF1a positive/negative.

We used Cohen's κ statistics to compare the value of each of the antibodies as probes capable of categorizing each case as expressing (i.e. positive) or not expressing (negative) nuclear HIF-1α. As reported in the Methods section, a "positive" designation required easily-detectable immunoreactivity with three out of five antibodies. A sample showing nuclear immunoreactivity using one or two antibodies would be designated as "negative". A high κ value compared to HIF-1α positive patients would indicate that a given antibody performed better in detecting positive cases when compared with lower κ value antibodies. The κ values (95% confidence intervals) for the different antibodies are presented in ranking order in Table 3. The antibody with the lowest kappa value was H1a67-Abcam. Birner et al. used this antibody in his IHC study and reported that HIF-1α overexpression had no impact on the prognosis [6]. To verify this, we performed a separate survival analysis using only the findings from this antibody (Figure 4) and the survival analysis was not significant, as Birner et al. had reported [6].

**Table 3:** Kappa values (κ value 95% confidence interval) of antibodies to HIF-1α used in this study*

**Antibody**	**Optimal dilution**	**Source**	**κ value (95% confidence interval)**
54/HIF-1α	1:20	BD Biosciences	0.754 (0.435–1.073)
Rabbit polyclonal	1:200	Univ. Thessaly	0.634 (0.368–0.901)
Rabbit Polyclonal	1:200	Santa Cruz	0.618 (0.282–0.955)
H1a67	1:75	Neomarkers	0.569 (0.287–0.851)
H1a67	1:200	Abcam	0.435 (0.126–0.744).

## Discussion

HIF-1α expression has been noted in many different tumor types and has been variably associated with adverse prognosis. In ovarian carcinomas, a role for HIF-1α as an adverse prognostic factor has been suggested but there are conflicting findings [10]. Data variation can be ascribed to many factors, including methodological differences or statistical discrepancies due to the small size of patient study groups. We observed that the frequency of nuclear expression of HIF-1α in benign tumours was lower than in borderline and ovarian cancer tumours, which is in agreement with previous findings [6,7,10] and supports the assertion that HIF-1α can be considered a hallmark of tumour progression in ovarian carcinomas.

The frequency of nuclear expression of HIF-1α in stage III carcinomas was higher than in stage I tumours, but this was not statistically significant. However two points must be emphasised. First we included only poorly differentiated serous carcinomas in the survival analysis of our study and second we observed high HIF-1α expression in almost 50% of the stage I cases (12/25). Nakayama et al., using RT-PCR, also reported that HIF-1α expression level was independent of clinical stage [8]. However, Osada et al. reported that HIF-1α nuclear expression was significantly higher in stage III and IV tumours than in those of stages I and II [10]. Different histological types of ovarian carcinoma were used in his study, and HIF-1α immunostaining was observed in only 12 of the 48 cases of stages I and II (25%). In agreement with our data, Osada et al. also reported higher expression in serous neoplasms reporting that the role of the HIF pathway in ovarian cancer might be different among tumour subtypes [10].

In the 55 patients with stage III, G3 serous carcinoma in this study, the overall survival of patients with tumours that stained strongly for HIF-1α was significantly shorter. This is in accordance with the recent in vivo IHC study by Osada et al. [10], but contradicts the previous studies by Birner et al. [6] and Nakayama et al. [8]. A possible explanation is the difference in adjuvant chemotherapy.

Since we noted that HIF-1α assessment can be antibody-dependent, a prudent future approach would be to use a panel of antibodies in order to increase the overall sensitivity of the immunoassay, at the same time maintaining acceptable levels of specificity as we did in our study. This could be achieved with the use of an affinity-purified polyclonal antibody and one or two well-characterised monoclonal antibodies. In future studies, additional validation of the immunoassay could be performed by including downstream targets of HIF-1α as their upregulation is due to HIF-1α pathway activation [4]. Alternatively, if there is an established correlation between HIF-1α expression and a clinical variable, the performance of the antibody could be tested by its potential to reproduce the aforementioned association.

Our results suggest that the difference in progress free interval is closely related to the chemoresponse of postoperative chemotherapy. One interpretation is that high HIF-1α-expressing suboptimally resected tumours are chemoresistant. It has been previously reported that expression of HIF-1α may be predictive of responsiveness to adjuvant therapy and radiotherapy [13-15]. Previous studies showed that tumour hypoxia could lead to chemoresistance directly, due to the lack of oxygen availability, and indirectly due to the alteration in gene expression and subsequent changes in angiogenesis and pH changes [16]. Since HIF-1α is the key molecule regulated by tumour hypoxia, HIF-1α deregulation in tumour cells may confer resistance in these cells [17,18]. However, it was recently reported using Western Blot analysis that HIF-1α-expressing ovarian tumours had a significantly higher rate of response to postoperative TC chemotherapy and exhibited significantly better survival [9]. The suggested explanation was that taxanes could be effective in down-regulating HIF-1α protein via effects on the microtubule cytoskeleton that are correlated with HIF-1α mRNA translation [19].

Cytoplasmic HIF-1α expression is more prominent in serous and endometrioid carcinoma, however this staining pattern is unexpected, since HIF-1α should translocate from the cytoplasm to the nucleus in order to activate its target genes within a short period of time, and accumulation of cytoplasmic HIF-1α should not be detected [20]. However, one study showed that there is a HIF-1α variant that is stable even in normoxia and does not translocate to the nucleus under hypoxic conditions [21]. This splice variant corresponds to the N-terminal part (aa 1–516) of the wild-type 826 aa long HIF-1a while most of the antibodies used in this study were raised against epitopes in the C-terminal part of HIF-1a. This plus the low relative expression of the variant make it rather unlikely that the observed cytoplasmic staining is due to the presence of this particular variant. Studies in other organ cancers have also reported that immunolocalization of HIF-1α is not limited inside the nucleus [22-24]. Our results, obtained by log-rank test, showed that the prognosis was poorer in patients with nuclear HIF-1α immunostaining. However, immunostaining for cytoplasmic HIF-1α was associated with shorter PFI and similar survival, but this was not statistically significant, which agrees with a recent study by Osada et al. [10].

In this respect it should be noted that as we reported previously nuclear localization of HIF-1α requires its modification by p42/44 MAPK [25] Therefore, exclusive nuclear immunostaining of HIF-1α could indicate an activated MAPK pathway and, subsequently, increased cellular proliferation.

We observed that the median PFI of HIF-1α positive patients was shorter but not statistically significant. However, in the subgroup of patients with suboptimally cytoreduced tumours, the PFI of patients with tumours that stained strongly for HIF-1α was significantly worse than that of patients with tumours that stained weakly or were negative for HIF-1α, and in those patients it proved to be an independent prognostic factor. This is in accordance with previous studies which showed that tumour hypoxia could lead to chemoresistance directly [16].

The HIF system arises as an important molecular target in the treatment of ovarian carcinoma. Staining the tumour tissue obtained from the primary laparotomy with HIF 1a antibodies could result in grouping them in positive or negative as in our study, HIF 1a positive patients could enter clinical trials using a number of agents that inhibit HIF-1α accumulation including topotecan [26], 2-methoxyestradiol [27,28] and the Hsp90 inhibitors [29,30]. Furthermore using the experimental models to test the efficacy of rapamycin in ovarian cancer treatment, a significant correlation between HIF-1α inhibition and VEGF down-regulation or increase of apoptosis has been demonstrated [31], and it was mentioned that rapamycin delays the tumour onset and progression [32]. Additional effects were found to be exerted when rapamycin is administered in combination with paclitaxel [31] and tamoxifen [33] and could also be investigated in clinical trials.

Clearly, additional studies are needed, however, we strongly support determination of HIF-1-α expression by immunohistochemistry in serous ovarian cancer for devising subgroups for individualized treatment regimens.

## Conclusion

HIF-1α expression has been noted in many tumours and it has been variably associated with adverse prognosis. Our report confirms the prognostic value of HIF-1α in serous ovarian cancer in a specific, albeit large, subset of patients. In addition, we show that this association is elusive, since it is not only methodology-related but it can be antibody-dependent. From our observations, nuclear HIF-1α expression might represent an important biological marker in the evaluation of the prognosis of patients with poorly differentiated serous ovarian carcinoma.

In our study, HIF-1α was an independent prognostic factor of survival and could be used to assist in decisions for adjuvant therapies.

Furthermore, in designing a HIF-1α-targeting clinical trial, it would be important to optimize the assessment of HIF-1α expression by using a panel of antibodies to accurately identify subgroups of ovarian carcinoma patients who could benefit from novel HIF-1α-inhibiting therapeutic strategies.

## Abbreviations

HIF: Hypoxia-inducible factor; HIF-1α: Hypoxia-Inducible Factor 1α; IHC: Immunohistochemistry; PFI: Progress free interval; TC: Taxol, Carbo; AUC: Area under the concentration curve

## Competing interests

The authors declare that they have no competing interests.

## Authors' contributions

AD designed the study and drafted the manuscript. He was involved as primary surgeon or first assistant in all the patients surgery, was responsible for patients management during their stay in the hospital and participated in the muldisciplinary meeting regarding adjuvant therapy and was responsible for their clinical follow up through the gynaecological oncology outpatients clinic. MI gave the pathology report in many cases, reviewed all the pathology reports for the purpose of the study and collected tumor specimens, organized the stainings, scored sections and contributed to the design of the study and the drafting of the manuscript. IM gave his technical assistance in the laboratory. GS organized the stainings, contributed to the design of the study and the drafting of the manuscript. IMM, conducted the statistical analyses, prepared figures and tables and helped draft the manuscript. IEM performed as primary surgeon patients surgery and contributed to the drafting of the manuscript. GK reviewed all the pathology reports for the purpose of the study, contributed to tumor specimens selection and scoring and organised the setup of the ovarian carcinoma database. He contributed to the design of the study and the drafting of the manuscript. All authors read and approved the final manuscript.

## Pre-publication history

The pre-publication history for this paper can be accessed here:


